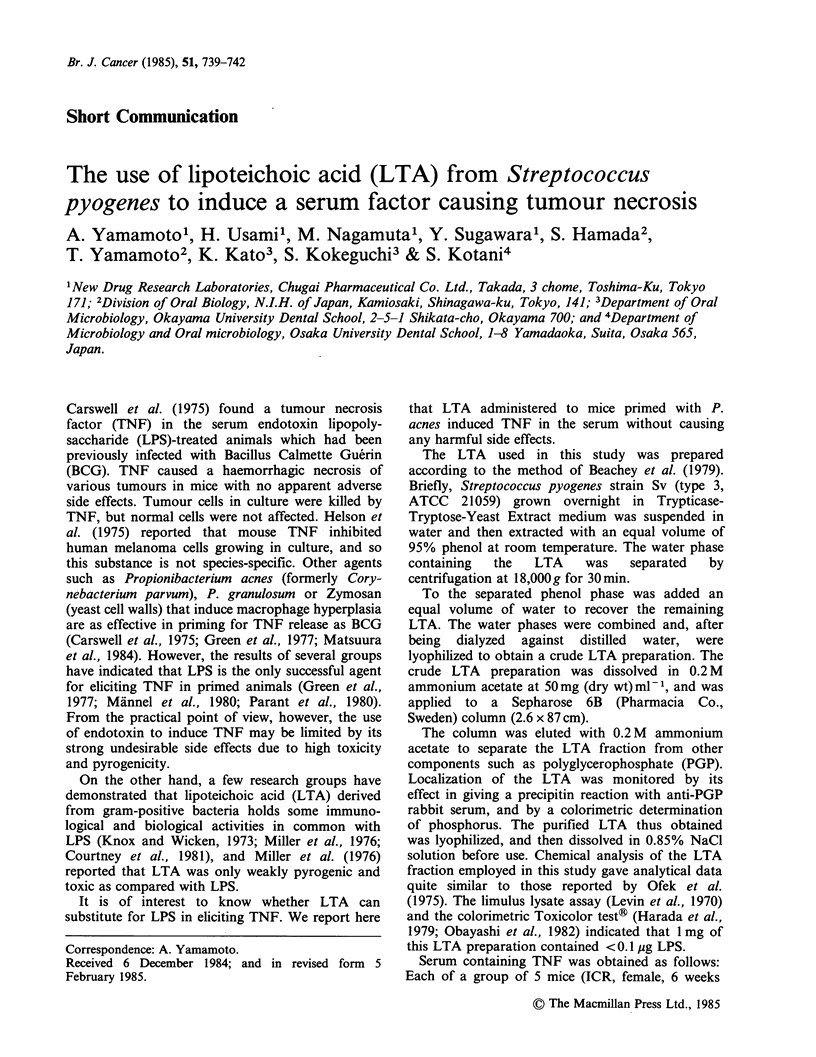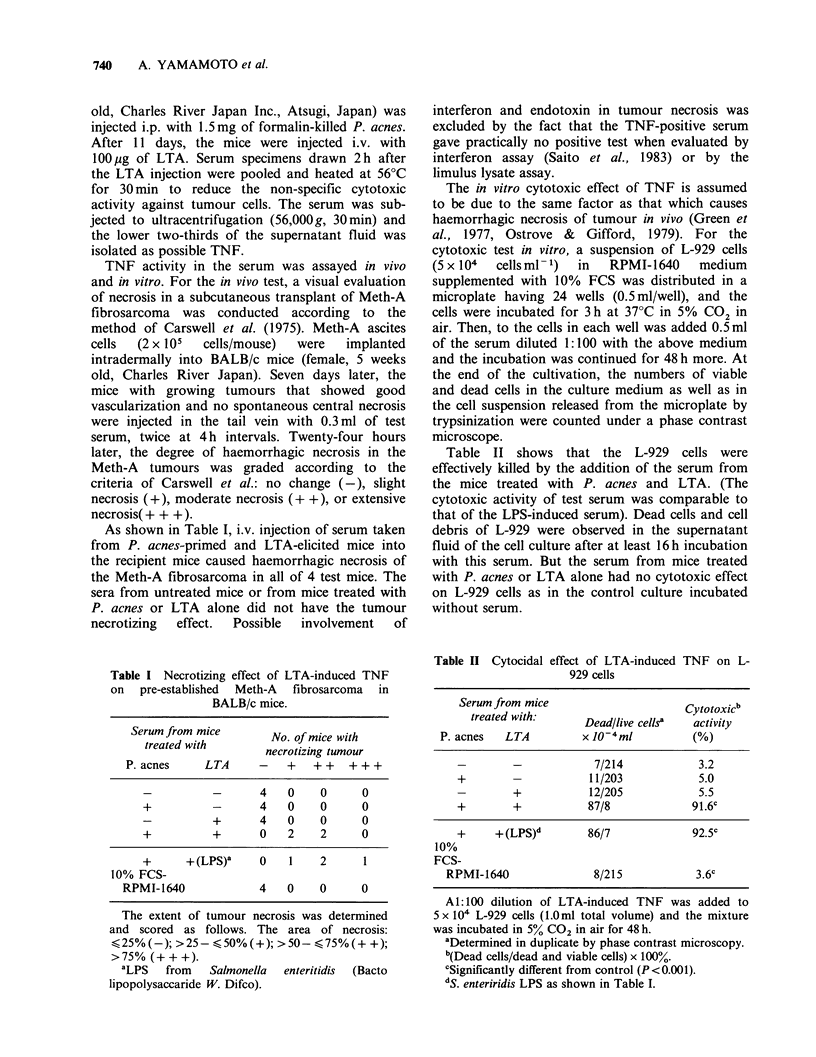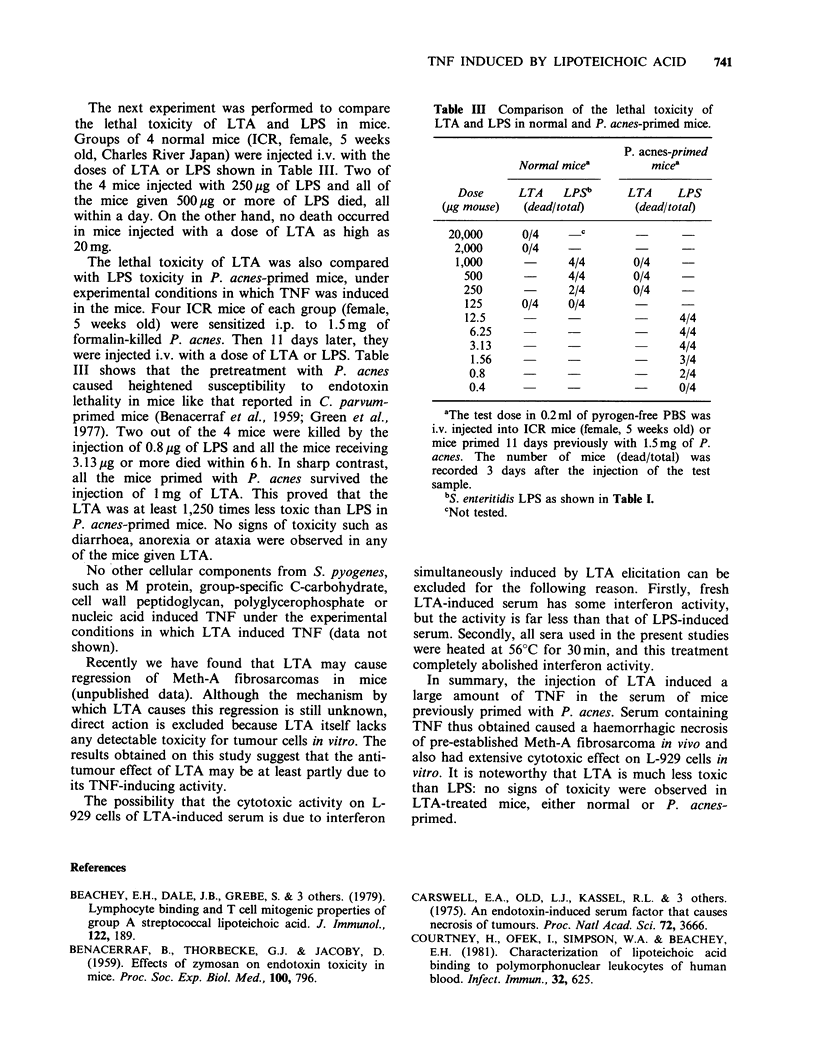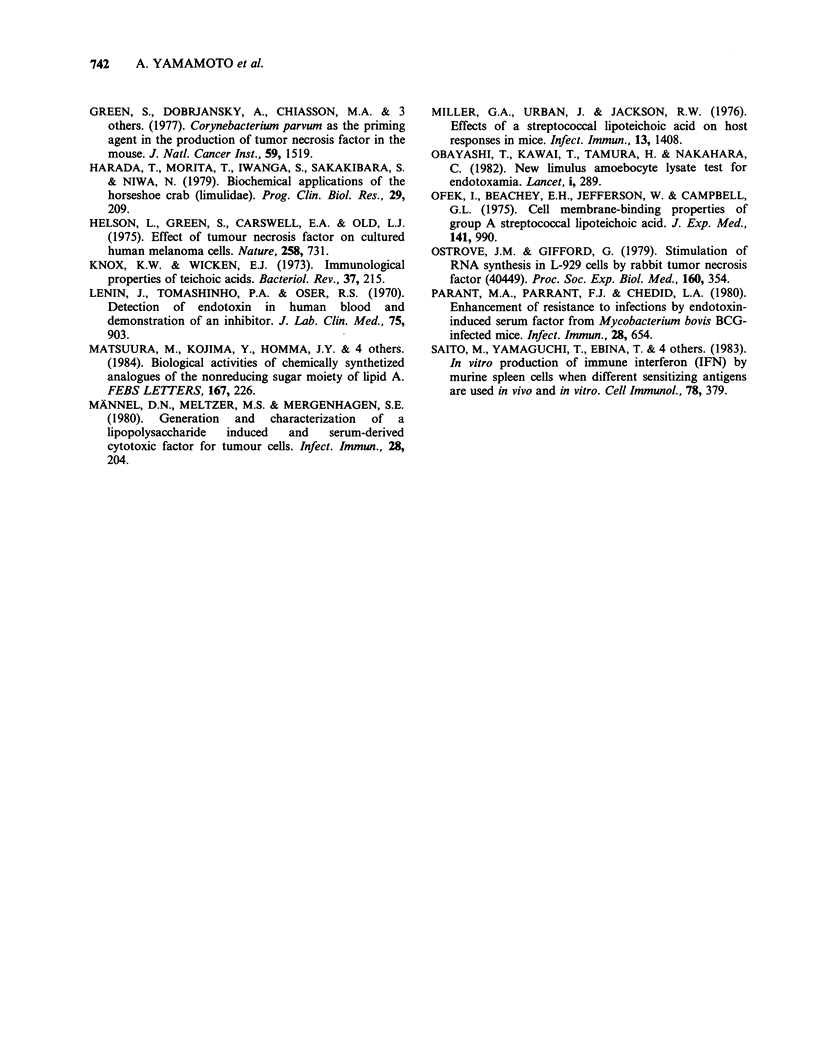# The use of lipoteichoic acid (LTA) from Streptococcus pyogenes to induce a serum factor causing tumour necrosis.

**DOI:** 10.1038/bjc.1985.112

**Published:** 1985-05

**Authors:** A. Yamamoto, H. Usami, M. Nagamuta, Y. Sugawara, S. Hamada, T. Yamamoto, K. Kato, S. Kokeguchi, S. Kotani


*Br. J. Cancer* (1985), **51**, 739–742

### Short Communication

### The use of lipoteichoic acid (LTA) from *Streptococcus pyogenes* to induce a serum factor causing tumour necrosis

A. Yamamoto[1], H. Usami[1], M. Nagamuta[1], Y. Sugawara[1], S. Hamada[2], T. Yamamoto[2], K. Kato[3], S. Kokeguchi[3] & S. Kotani[4]

[1]*New Drug Research Laboratories, Chugai Pharmaceutical Co. Ltd., Takada, 3 chome, Toshima-Ku, Tokyo 171;* [2]*Division of Oral Biology, N.I.H. of Japan, Kamiosaki, Shinagawa-ku, Tokyo, 141;* [3]*Department of Oral Microbiology, Okayama University Dental School, 2-5-1 Shikata-cho, Okayama 700; and* [4]*Department of Microbiology and Oral microbiology, Osaka University Dental School, 1-8 Yamadaoka, Suita, Osaka 565, Japan.*

Carswell *et al.* (1975) found a tumour necrosis factor (TNF) in the serum endotoxin lipopolysaccharide (LPS)-treated animals which had been previously infected with Bacillus Calmette Guérin (BCG). TNF caused a haemorrhagic necrosis of various tumours in mice with no apparent adverse side effects. Tumour cells in culture were killed by TNF, but normal cells were not affected. Helson *et al.* (1975) reported that mouse TNF inhibited human melanoma cells growing in culture, and so this substance is not species-specific. Other agents such as *Propionibacterium acnes* (formerly *Corynebacterium parvum*), *P. granulosum* or Zymosan (yeast cell walls) that induce macrophage hyperplasia are as effective in priming for TNF release as BCG (Carswell *et al.*, 1975; Green *et al.*, 1977; Matsuura *et al.*, 1984). However, the results of several groups have indicated that LPS is the only successful agent for eliciting TNF in primed animals (Green *et al.*, 1977; Männel *et al.*, 1980; Parant *et al.*, 1980). From the practical point of view, however, the use of endotoxin to induce TNF may be limited by its strong undesirable side effects due to high toxicity and pyrogenicity.

On the other hand, a few research groups have demonstrated that lipoteichoic acid (LTA) derived from gram-positive bacteria holds some immunological and biological activities in common with LPS (Knox and Wicken, 1973; Miller *et al.*, 1976; Courtney *et al.*, 1981), and Miller *et al.* (1976) reported that LTA was only weakly pyrogenic and toxic as compared with LPS.

It is of interest to know whether LTA can substitute for LPS in eliciting TNF. We report here that LTA administered to mice primed with *P. acnes* induced TNF in the serum without causing any harmful side effects.

The LTA used in this study was prepared according to the method of Beachey *et al.* (1979). Briefly, *Streptococcus pyogenes* strain Sv (type 3, ATCC 21059) grown overnight in Trypticase-Tryptose-Yeast Extract medium was suspended in water and then extracted with an equal volume of 95% phenol at room temperature. The water phase containing the LTA was separated by centrifugation at 18,000 *g* for 30 min.

To the separated phenol phase was added an equal volume of water to recover the remaining LTA. The water phases were combined and, after being dialyzed against distilled water, were lyophilized to obtain a crude LTA preparation. The crude LTA preparation was dissolved in 0.2 M ammonium acetate at 50 mg (dry wt) $ml^{-1}$, and was applied to a Sepharose 6B (Pharmacia Co., Sweden) column (2.6 × 87 cm).

The column was eluted with 0.2 M ammonium acetate to separate the LTA fraction from other components such as polyglycerophosphate (PGP). Localization of the LTA was monitored by its effect in giving a precipitin reaction with anti-PGP rabbit serum, and by a colorimetric determination of phosphorus. The purified LTA thus obtained was lyophilized, and then dissolved in 0.85% NaCl solution before use. Chemical analysis of the LTA fraction employed in this study gave analytical data quite similar to those reported by Ofek *et al.* (1975). The limulus lysate assay (Levin *et al.*, 1970) and the colorimetric Toxicolor test® (Harada *et al.*, 1979; Obayashi *et al.*, 1982) indicated that 1 mg of this LTA preparation contained $<0.1\,\mu g$ LPS.

Serum containing TNF was obtained as follows: Each of a group of 5 mice (ICR, female, 6 weeks

Correspondence: A. Yamamoto.
Received 6 December 1984; and in revised form 5 February 1985.

© The Macmillan Press Ltd., 1985

old, Charles River Japan Inc., Atsugi, Japan) was injected i.p. with 1.5 mg of formalin-killed *P. acnes*. After 11 days, the mice were injected i.v. with 100 $\mu$g of LTA. Serum specimens drawn 2 h after the LTA injection were pooled and heated at 56°C for 30 min to reduce the non-specific cytotoxic activity against tumour cells. The serum was subjected to ultracentrifugation (56,000 *g*, 30 min) and the lower two-thirds of the supernatant fluid was isolated as possible TNF.

TNF activity in the serum was assayed *in vivo* and *in vitro*. For the *in vivo* test, a visual evaluation of necrosis in a subcutaneous transplant of Meth-A fibrosarcoma was conducted according to the method of Carswell *et al.* (1975). Meth-A ascites cells ($2 \times 10^5$ cells/mouse) were implanted intradermally into BALB/c mice (female, 5 weeks old, Charles River Japan). Seven days later, the mice with growing tumours that showed good vascularization and no spontaneous central necrosis were injected in the tail vein with 0.3 ml of test serum, twice at 4 h intervals. Twenty-four hours later, the degree of haemorrhagic necrosis in the Meth-A tumours was graded according to the criteria of Carswell *et al.*: no change (−), slight necrosis (+), moderate necrosis (+ +), or extensive necrosis(+ + +).

As shown in Table I, i.v. injection of serum taken from *P. acnes*-primed and LTA-elicited mice into the recipient mice caused haemorrhagic necrosis of the Meth-A fibrosarcoma in all of 4 test mice. The sera from untreated mice or from mice treated with *P. acnes* or LTA alone did not have the tumour necrotizing effect. Possible involvement of interferon and endotoxin in tumour necrosis was excluded by the fact that the TNF-positive serum gave practically no positive test when evaluated by interferon assay (Saito *et al.*, 1983) or by the limulus lysate assay.

The *in vitro* cytotoxic effect of TNF is assumed to be due to the same factor as that which causes haemorrhagic necrosis of tumour *in vivo* (Green *et al.*, 1977, Ostrove & Gifford, 1979). For the cytotoxic test *in vitro*, a suspension of L-929 cells ($5 \times 10^4$ cells $ml^{-1}$) in RPMI-1640 medium supplemented with 10% FCS was distributed in a microplate having 24 wells (0.5 ml/well), and the cells were incubated for 3 h at 37°C in 5% $CO_2$ in air. Then, to the cells in each well was added 0.5 ml of the serum diluted 1:100 with the above medium and the incubation was continued for 48 h more. At the end of the cultivation, the numbers of viable and dead cells in the culture medium as well as in the cell suspension released from the microplate by trypsinization were counted under a phase contrast microscope.

Table II shows that the L-929 cells were effectively killed by the addition of the serum from the mice treated with *P. acnes* and LTA. (The cytotoxic activity of test serum was comparable to that of the LPS-induced serum). Dead cells and cell debris of L-929 were observed in the supernatant fluid of the cell culture after at least 16 h incubation with this serum. But the serum from mice treated with *P. acnes* or LTA alone had no cytotoxic effect on L-929 cells as in the control culture incubated without serum.

**Table I** Necrotizing effect of LTA-induced TNF on pre-established Meth-A fibrosarcoma in BALB/c mice.

| *Serum from mice treated with* | | *No. of mice with necrotizing tumour* | | | |
|---|---|---|---|---|---|
| P. acnes | *LTA* | − | + | + + | + + + |
| − | − | 4 | 0 | 0 | 0 |
| + | − | 4 | 0 | 0 | 0 |
| − | + | 4 | 0 | 0 | 0 |
| + | + | 0 | 2 | 2 | 0 |
| + | +(LPS)[a] | 0 | 1 | 2 | 1 |
| 10% FCS-RPMI-1640 | | 4 | 0 | 0 | 0 |

The extent of tumour necrosis was determined and scored as follows. The area of necrosis: ≤25% (−); >25 − ≤50% (+); >50 − ≤75% (+ +); >75% (+ + +).

[a]LPS from *Salmonella enteritidis* (Bacto lipopolysaccaride *W*. Difco).

**Table II** Cytocidal effect of LTA-induced TNF on L-929 cells

| *Serum from mice treated with:* P. acnes | *LTA* | *Dead/live cells*[a] $\times 10^{-4}$ *ml* | *Cytotoxic*[b] *activity* (%) |
|---|---|---|---|
| − | − | 7/214 | 3.2 |
| + | − | 11/203 | 5.0 |
| − | + | 12/205 | 5.5 |
| + | + | 87/8 | 91.6[c] |
| + | +(LPS)[d] | 86/7 | 92.5[c] |
| 10% FCS-RPMI-1640 | | 8/215 | 3.6[c] |

A1:100 dilution of LTA-induced TNF was added to $5 \times 10^4$ L-929 cells (1.0 ml total volume) and the mixture was incubated in 5% $CO_2$ in air for 48 h.

[a]Determined in duplicate by phase contrast microscopy.

[b](Dead cells/dead and viable cells) × 100%.

[c]Significantly different from control ($P < 0.001$).

[d]*S. enteriridis* LPS as shown in Table I.

The next experiment was performed to compare the lethal toxicity of LTA and LPS in mice. Groups of 4 normal mice (ICR, female, 5 weeks old, Charles River Japan) were injected i.v. with the doses of LTA or LPS shown in Table III. Two of the 4 mice injected with 250 $\mu$g of LPS and all of the mice given 500 $\mu$g or more of LPS died, all within a day. On the other hand, no death occurred in mice injected with a dose of LTA as high as 20 mg.

The lethal toxicity of LTA was also compared with LPS toxicity in *P. acnes*-primed mice, under experimental conditions in which TNF was induced in the mice. Four ICR mice of each group (female, 5 weeks old) were sensitized i.p. to 1.5 mg of formalin-killed *P. acnes*. Then 11 days later, they were injected i.v. with a dose of LTA or LPS. Table III shows that the pretreatment with *P. acnes* caused heightened susceptibility to endotoxin lethality in mice like that reported in *C. parvum*-primed mice (Benacerraf *et al.*, 1959; Green *et al.*, 1977). Two out of the 4 mice were killed by the injection of 0.8 $\mu$g of LPS and all the mice receiving 3.13 $\mu$g or more died within 6 h. In sharp contrast, all the mice primed with *P. acnes* survived the injection of 1 mg of LTA. This proved that the LTA was at least 1,250 times less toxic than LPS in *P. acnes*-primed mice. No signs of toxicity such as diarrhoea, anorexia or ataxia were observed in any of the mice given LTA.

No other cellular components from *S. pyogenes*, such as M protein, group-specific C-carbohydrate, cell wall peptidoglycan, polyglycerophosphate or nucleic acid induced TNF under the experimental conditions in which LTA induced TNF (data not shown).

Recently we have found that LTA may cause regression of Meth-A fibrosarcomas in mice (unpublished data). Although the mechanism by which LTA causes this regression is still unknown, direct action is excluded because LTA itself lacks any detectable toxicity for tumour cells *in vitro*. The results obtained on this study suggest that the anti-tumour effect of LTA may be at least partly due to its TNF-inducing activity.

The possibility that the cytotoxic activity on L-929 cells of LTA-induced serum is due to interferon simultaneously induced by LTA elicitation can be excluded for the following reason. Firstly, fresh LTA-induced serum has some interferon activity, but the activity is far less than that of LPS-induced serum. Secondly, all sera used in the present studies were heated at 56°C for 30 min, and this treatment completely abolished interferon activity.

In summary, the injection of LTA induced a large amount of TNF in the serum of mice previously primed with *P. acnes*. Serum containing TNF thus obtained caused a haemorrhagic necrosis of pre-established Meth-A fibrosarcoma *in vivo* and also had extensive cytotoxic effect on L-929 cells *in vitro*. It is noteworthy that LTA is much less toxic than LPS: no signs of toxicity were observed in LTA-treated mice, either normal or *P. acnes*-primed.

**Table III** Comparison of the lethal toxicity of LTA and LPS in normal and *P. acnes*-primed mice.

| | *Normal mice*[a] | | P. acnes-*primed mice*[a] | |
|---|---|---|---|---|
| *Dose* (*μg mouse*) | *LTA* | *LPS*[b] | *LTA* | *LPS* |
| | (*dead/total*) | | (*dead/total*) | |
| 20,000 | 0/4 | —[c] | — | — |
| 2,000 | 0/4 | — | — | — |
| 1,000 | — | 4/4 | 0/4 | — |
| 500 | — | 4/4 | 0/4 | — |
| 250 | — | 2/4 | 0/4 | — |
| 125 | 0/4 | 0/4 | — | — |
| 12.5 | — | — | — | 4/4 |
| 6.25 | — | — | — | 4/4 |
| 3.13 | — | — | — | 4/4 |
| 1.56 | — | — | — | 3/4 |
| 0.8 | — | — | — | 2/4 |
| 0.4 | — | — | — | 0/4 |

[a]The test dose in 0.2 ml of pyrogen-free PBS was i.v. injected into ICR mice (female, 5 weeks old) or mice primed 11 days previously with 1.5 mg of *P. acnes*. The number of mice (dead/total) was recorded 3 days after the injection of the test sample.
[b]*S. enteritidis* LPS as shown in **Table I.**
[c]Not tested.

### References

BEACHEY, E.H., DALE, J.B., GREBE, S. & 3 others. (1979). Lymphocyte binding and T cell mitogenic properties of group A streptococcal lipoteichoic acid. *J. Immunol.*, **122,** 189.

BENACERRAF, B., THORBECKE, G.J. & JACOBY, D. (1959). Effects of zymosan on endotoxin toxicity in mice. *Proc. Soc. Exp. Biol. Med.*, **100,** 796.

CARSWELL, E.A., OLD, L.J., KASSEL, R.L. & 3 others. (1975). An endotoxin-induced serum factor that causes necrosis of tumours. *Proc. Natl Acad. Sci.* **72,** 3666.

COURTNEY, H., OFEK, I., SIMPSON, W.A. & BEACHEY, E.H. (1981). Characterization of lipoteichoic acid binding to polymorphonuclear leukocytes of human blood. *Infect. Immun.*, **32,** 625.

GREEN, S., DOBRJANSKY, A., CHIASSON, M.A. & 3 others. (1977). *Corynebacterium parvum* as the priming agent in the production of tumor necrosis factor in the mouse. *J. Natl. Cancer Inst.*, **59**, 1519.

HARADA, T., MORITA, T., IWANGA, S., SAKAKIBARA, S. & NIWA, N. (1979). Biochemical applications of the horseshoe crab (limulidae). *Prog. Clin. Biol. Res.*, **29**, 209.

HELSON, L., GREEN, S., CARSWELL, E.A. & OLD, L.J. (1975). Effect of tumour necrosis factor on cultured human melanoma cells. *Nature*, **258**, 731.

KNOX, K.W. & WICKEN, E.J. (1973). Immunological properties of teichoic acids. *Bacteriol. Rev.*, **37**, 215.

LENIN, J., TOMASHINHO, P.A. & OSER, R.S. (1970). Detection of endotoxin in human blood and demonstration of an inhibitor. *J. Lab. Clin. Med.*, **75**, 903.

MATSUURA, M., KOJIMA, Y., HOMMA, J.Y. & 4 others. (1984). Biological activities of chemically synthetized analogues of the nonreducing sugar moiety of lipid A. *FEBS LETTERS*, **167**, 226.

MÄNNEL, D.N., MELTZER, M.S. & MERGENHAGEN, S.E. (1980). Generation and characterization of a lipopolysaccharide induced and serum-derived cytotoxic factor for tumour cells. *Infect. Immun.*, **28**, 204.

MILLER, G.A., URBAN, J. & JACKSON, R.W. (1976). Effects of a streptococcal lipoteichoic acid on host responses in mice. *Infect. Immun.*, **13**, 1408.

OBAYASHI, T., KAWAI, T., TAMURA, H. & NAKAHARA, C. (1982). New limulus amoebocyte lysate test for endotoxamia. *Lancet*, **i**, 289.

OFEK, I., BEACHEY, E.H., JEFFERSON, W. & CAMPBELL, G.L. (1975). Cell membrane-binding properties of group A streptococcal lipoteichoic acid. *J. Exp. Med.*, **141**, 990.

OSTROVE, J.M. & GIFFORD, G. (1979). Stimulation of RNA synthesis in L-929 cells by rabbit tumor necrosis factor (40449). *Proc. Soc. Exp. Biol. Med.*, **160**, 354.

PARANT, M.A., PARRANT, F.J. & CHEDID, L.A. (1980). Enhancement of resistance to infections by endotoxin-induced serum factor from *Mycobacterium bovis* BCG-infected mice. *Infect. Immun.*, **28**, 654.

SAITO, M., YAMAGUCHI, T., EBINA, T. & 4 others. (1983). *In vitro* production of immune interferon (IFN) by murine spleen cells when different sensitizing antigens are used *in vivo* and *in vitro*. *Cell Immunol.*, **78**, 379.